# Does education of primary care professionals promote patient self-management and improve outcomes in chronic disease? An updated systematic review

**DOI:** 10.3399/BJGPO.2020.0186

**Published:** 2021-04-21

**Authors:** Claire Collins, Gillian Doran, Patricia Patton, Roisin Fitzgerald, Andree Rochfort

**Affiliations:** 1 Director of Research, Irish College of General Practitioners, Dublin, Ireland; 2 Head Librarian, Irish College of General Practitioners, Dublin, Ireland; 3 Librarian, Irish College of General Practitioners, Dublin, Ireland; 4 Research Assistant, Irish College of General Practitioners, Dublin, Ireland; 5 Director, Quality Improvement & Doctors Health Programme, Irish College of General Practitioners, Dublin, Ireland

**Keywords:** self-management, patient empowerment, patient participation, primary health care, chronic conditions

## Abstract

**Background:**

Primary care has a vital role in supporting patient autonomy to enable people with long-term conditions to manage their own health and wellness. Evidence is needed on whether education and training of health professionals helps support patient self-management and improves outcomes. The authors' first systematic review included only two articles showing patient outcomes following health professional training for promoting patient self-management.

**Aim:**

To present an updated review undertaken from September 2013 to August 2018.

**Design & setting:**

A systematic review was undertaken using the PRISMA guidelines, following the methodology of the first review and is outlined in the PROSPERO registered protocol.

**Method:**

Six databases were searched — Cochrane Library, PubMed, ERIC, Embase, Cumulative Index to Nursing and Allied Health Literature (CINAHL), and PsycINFO — in addition to web searches, hand searches, and bibliographies for articles published from 1 September 2013 to 31 August 2018.

**Results:**

The updated systematic review showed more evidence is now available with 18 articles in the 5-year period from the 4284 abstracts located. Twelve of these articles showed a difference between intervention and control groups. Of the 18 articles identified, 11 were assessed as having a low risk of bias and five overall were rated of weak quality. The educational interventions with health professionals spanned a range of techniques and modalities, and many incorporated multiple interventions including patient components. There may be a lack of adoption owing to several challenges, including that complex interventions may not be delivered as planned and are difficult to assess, and owing to patient engagement and the need for ongoing follow-up.

**Conclusion:**

More high-quality research is needed on what methods work best, for which patients, and for what clinical conditions in the primary care setting. The practical implications of training healthcare professionals require specific attention.

## How this fits in

Despite the vast literature on patient self-management, evidence on the association between training of health professionals in patient self-management and measured health outcomes was rare before and up to 2 years after its incorporation into the World Organization of Family Doctors (WONCA) Europe definition of general practice. Since the authors' previous systematic review, more published evidence is available to review (September 2013 to August 2018), which suggests a benefit to patient health outcomes and behaviour following health professional education. Interventions that include multiple aspects, follow-up, and patient-centred components are more likely to be successful; however, the implications for delivery and uptake in primary care need to be considered.

## Introduction

The World Health Organization (WHO) defines chronic conditions as those that encompass disability and disease that people ‘live with’ for extended periods of time.^1^ The Chronic Care Model^2^ is an internationally accepted model for the management of non-communicable diseases and specifies self-management support as a key component. The concept of patient empowerment for self-management was introduced into the WONCA Europe definition of general practice in 2011.^3^ Patient empowerment is a core concept of patient-centred care^3,4^ — a widely called for concept^5^ — and has been shown to be central to the improvement of self-management programmes,^6,7^ as has the need to recognise the phases of transformation for individual patients.^8^

Some studies demonstrate the benefit of self-management support^9–18^ for people with chronic conditions; however, it is also reported that patients with chronic conditions tend not to respond as well to lifestyle interventions.^19^ Primary care has a key role in supporting patient autonomy to enable patients to develop expertise in managing their own health and wellness.^20^ This support has been identified as a potentially impactful avenue,^21^ with education and training noted as potential ways of engaging primary care clinicians in patient self-management support.^22^ However, it is also recognised that visits in primary care may be brief and that low levels of readiness to change may exist among patients.^23^

The authors' first systematic review of 7533 abstracts published before September 2013 included only two articles showing patient outcomes following health professional training for promoting patient self-management.^24^ Both included articles suggested that primary care health professionals can help to harness patients’ capacity to contribute to improvement of their own health outcomes. However, the review concluded the evidence was very limited on measured patient health outcomes.

The central focus of this project was to update that review and to systematically review the evidence from September 2013 to August 2018.

## Method

A systematic review was undertaken using the PRISMA guidelines^25^ and follows the methodology outlined in the PROSPERO registered protocol.^26^

### Sourcing information

Two specialist subject librarians assisted in the development of the search strategy, which replicated the strategy used in the first review and was designed to identify internationally recognised terminology in peer-reviewed journals. Full details of this strategy are available in the published protocol.^26^ Six databases were searched — Cochrane Library, PubMed, ERIC, Embase, CINAHL, and PsycINFO — in addition to web searches, hand searches, and bibliographies. Articles published from 1 September 2013 to 31 August 2018 were included in the review, with the search conducted by two authors. The full search terms have been previously published.^24^

### Selection criteria

Studies with the following designs were included: systematic reviews, meta-analysis, randomised controlled trials (RCTs), controlled clinical trials, interrupted time series, and controlled before-and-after studies. Participants were physicians in primary care settings, other clinicians in primary care settings, and patients aged ≥18 years with chronic conditions in primary care settings. Included interventions had an educational focus designed to train primary care clinicians to support patient self-management. This review was concerned with all chronic conditions as they occur generically in the primary care setting, rather than focusing on any one specific chronic condition. Only articles including reference to patient outcomes, measured using validated measurement scales, were included. The primary patient outcome was change in patients’ self-management behaviours. The secondary outcomes were changes in physical health measures; health behaviours, including medical adherence and compliance; service utilisation; psychological health; psychosocial function, for example, quality of life; physical functioning; and knowledge. The eligibility of studies was determined using the inclusion and exclusion criteria listed in the registered protocol and shown in Table 1.

**Table 1.** Inclusion and exclusion criteria

**Inclusion criteria**	**Exclusion criteria**	**Exclusion code**
English articles	Non-English articles	Eng
Adults (aged ≥18 years)	Study population aged <18 years	Age
Primary care or community	Secondary care or hospital	Not PC
Chronic conditions, chronic illness, chronic disease, non-communicable disease (NCD)	Acute conditions	Acute
Study type: systematic reviews, meta-analysis, RCTs, controlled clinical trials, interrupted time series, controlled before-and-after studies	Study type: qualitative studies, populations studies, surveys, cross sectional, uncontrolled before-and-after studies (cohort)	Study
Education and training of primary care health professionals for patient education in promoting change, behaviour change, lifestyle change, patient engagement, patient empowerment, motivational skills, patient collaboration, patient adherence and compliance, patient self-management, decision making, and patient problem-solving	Not education or training of healthcare professionals	Int
	Not primary care health professionals	Pop
	Primary outcome measures not included	Out
	Direct patient education only	Edu
Continuing education, CME, lifelong learning, or evidence-based medicine	Guideline adherence, clinical performance (no educational component involved)	Guid
All studies published from 1 September 2013 to 31 August 2018	Any article outside this timeframe	Date
	Organisational interventions	Org
	Financial changes and incentives	Fi
	Regulatory interventions	Reg

CME = continuing medical education. PC = primary care. RCTs = randomised controlled trials.

### Data extraction

All abstracts were reviewed using the RefWorks package to categorise the abstracts identified by the search. The initial review of abstracts was undertaken by one author, with 10% of abstracts re-checked by two other authors. The full-text articles of all those considered to be of possible relevance to the systematic review were read independently by two authors, and categorised using the same exclusion reasons. Disagreements were reviewed by another author. The quality assessment and extraction of thematic content of the final list of articles applicable to the systematic review question were considered by the two authors who read the full-text articles.

### Risk of bias and quality assessment

The risk of bias was assessed using the Cochrane Collaboration’s tool for randomised trials.^27^ It assessed the overall quality of individual studies using the Quality of Assessment Tool for Quantitative Studies.^28^ The risk of bias tool covers six domains of bias (selection bias, performance bias, detection bias, attrition bias, reporting bias, and other bias) with assessments on one or more aspects within each.^27^ Reviewers rated six components of quality (selection bias, study design, confounders, blinding, data collection methods, and withdrawals and dropouts) leading to an overall methodological quality rating for each study of strong (no weak ratings), moderate (one weak rating), or weak (two or more weak ratings).^28^ Reviewers resolved rating disagreements through discussion.

### Data synthesis

A narrative data synthesis was performed as per the authors' original protocol^26^ and the first systematic review^24^ on this topic.

## Results

### Study review and selection

**Figure 1.**
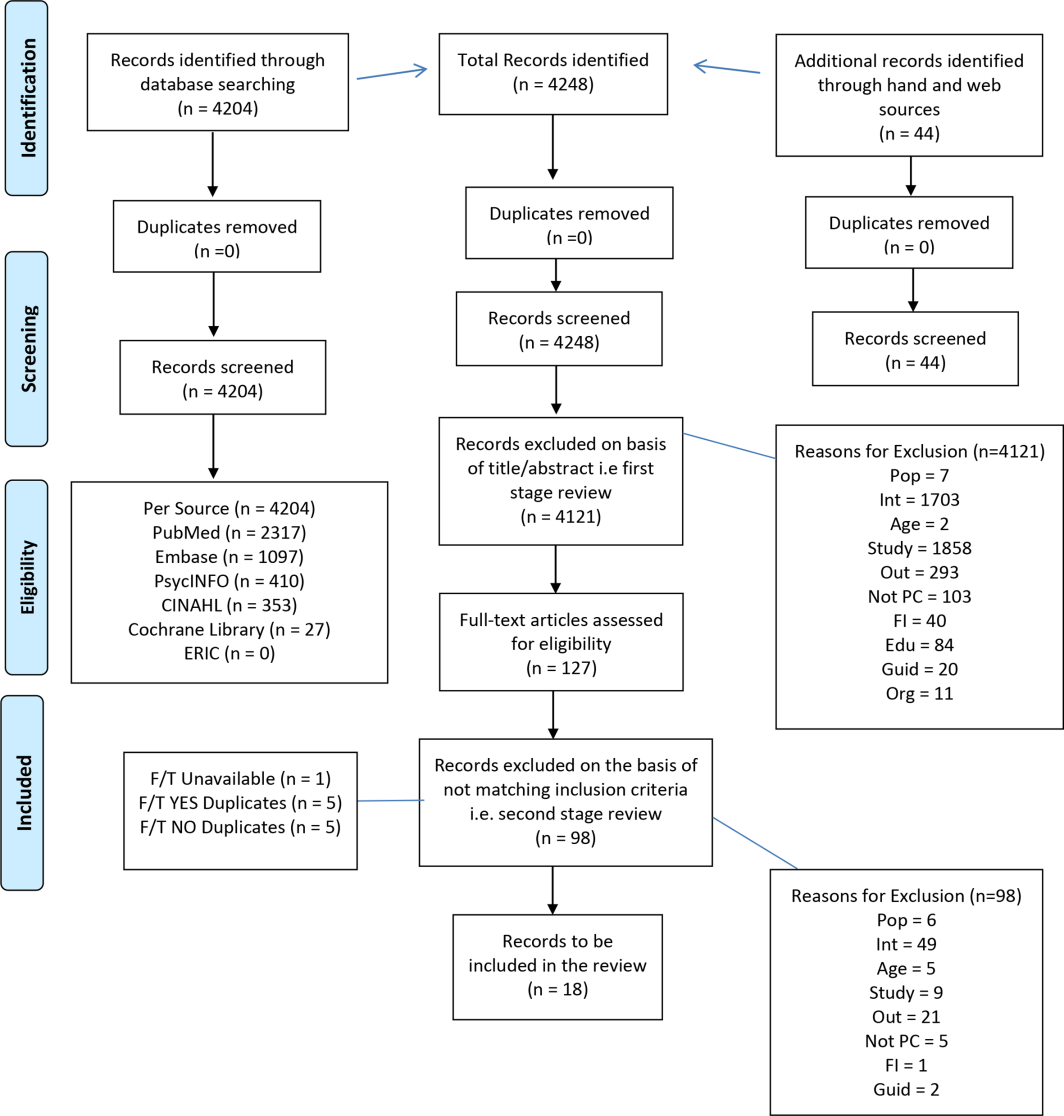
PRISMA flow diagram (see Table 1 for definition of exclusion code). F/T = full-text.

Overall, 4284 abstracts were found and 127 full-text articles were retrieved and read (Figure 1). Following the second-stage review, 18 articles reported patient outcomes and were included in the systematic review (see Supplementary Table S1).

All 18 articles were RCTs of educational interventions with primary care health professionals and examined their impact on patient outcome measures.^29–46^ The primary outcome of this review is the effectiveness of educational interventions with health professionals in terms of patient outcomes. Twelve of the 18 articles observed a significant difference between patient outcomes of those attending the intervention and control practices.^29,31–33,35–37,41,43–46^ Eleven articles overall — seven^31,33,36,37,41,43,44^ of the 12 articles showing an effect and four^30,34,38,40^ of the six articles not showing an effect — were considered to have a low risk of bias^27^ (see Supplementary Table S2). Among the seven articles that showed a difference in patient outcomes and had a low risk of bias, all were rated as moderate or strong in terms of the quality assessment (Table 2).^28^ Among the four trials that did not show significant differences in outcomes and were considered to have a low risk of bias, two were considered of weak quality and two of moderate quality.

**Table 2.** Quality rating of included papers

**First author**	GlobalScore	Selection bias	Study design	Confounders	Blinding	Data collection methods	Withdrawals and drop outs
Tobe^29^	weak	weak	mod	weak	weak	strong	mod
Kruis^30^	mod	weak	strong	strong	mod	strong	mod
Vicens^31^	strong	strong	strong	strong	weak	strong	strong
Keeley^32^	strong	mod	strong	strong	strong	strong	strong
Kristoffersen^33^	mod	weak	strong	strong	strong	strong	strong
van Dijk-de Vries^34^	weak	weak	strong	weak	mod	strong	mod
Racic^35^	strong	strong	strong	strong	mod	strong	strong
Vicens^36^	strong	strong	strong	strong	strong	strong	strong
Kristoffersen^37^	mod	weak	strong	strong	strong	strong	strong
Zwar^38^	weak	weak	strong	strong	strong	mod	strong
van Lieshout^39^	mod	weak	strong	strong	mod	strong	strong
Vaillant-Roussel^40^	mod	weak	strong	mod	mod	strong	mod
Griffiths^41^	strong	strong	strong	mod	mod	strong	mod
Eikelenboom^42^	strong	strong	strong	strong	mod	strong	strong
Ramli^43^	mod	strong	strong	strong	weak	strong	strong
Keeley^44^	mod	weak	strong	strong	mod	strong	strong
Kristoffersen^45^	weak	weak	strong	strong	weak	strong	strong
Baldeón^46^	weak	weak	strong	weak	mod	strong	mod

mod = moderate.

All but two RCTs included condition homogeneous patients (those with diabetes, at risk of or with cardiovasular disease [CVD], asthma, chronic obstructive pulmonary disease [COPD], depression, or chronic headache). One study^42^ included patients with at least one chronic condition (diabetes, [risk of] CVD, asthma, or COPD) and one study^31,36^ included patients taking benzodiazepines daily for 6 months (including those with psychotic disorders, severe personality disorder, alcohol or illicit drug abuse, anxiety or depression in hospital, or being treated by a psychiatrist). Some studies reported multiple follow-up time points in one article,^30,38,40,41,44^ while other studies reported these in separate articles.^31,33,36,37,45^ Vicens *et al* reported on follow-up at 12 months and 16 months,^31,36^ and Kristoffersen *et al* reported at 3 months, 6 months, and an average of 16-months follow-up.^33,37,45^ Follow-up time varied across studies, from 1.5 months to 36 months among studies achieving differences between intervention and controls, and 3 months to 24 months among studies not showing differences in primary and/or secondary outcome measures.

The educational interventions with health professionals spanned a range of techniques and modalities, and many incorporated multiple interventions including patient components. None of the studies separated the impact of different intervention elements. Limited generalisability was a factor for all studies.

Successful programmes concluded that the need for ongoing patient follow-up and patient feedback is a time-consuming factor.^31,36,44^ However, a focus on person-centred care with individualised care plans and/or recording of lifestyle goals in the patient medical record were noted factors in some successful studies.^29,31,36,41,46^ One study surmised that a less time-consuming structured intervention with a written individualised stepped-dose reduction plan is as effective in primary care as a more complex intervention involving follow-up visits.^31^

There may be lack of adoption owing to several challenges, including that complex interventions may not be delivered as planned,^31,32,36^ often owing to workload implications,^31,36,40^ because of high dropout rates and low study integrity.^38,40,42^ Additionally, changes specifically owing to the interventions are sometimes difficult to assess.^44^ One study showed a positive impact of the intervention after 3-years follow-up to be 1.5 times more effective than usual care despite time and workload constraints.^36^ Booster training was included in some of the successful interventions.^43,44^ Cost-effectiveness analyses should form a part of all future evaluations according to one study,^41^ given the intensity of the interventions and evaluations required.

A focus on person-centred care where the care delivered is aligned to patients’ needs and expectations and is interlinked to chronic disease management, increases the effectiveness of intervention programmes.^46^ Low uptake of some of the patient interventions, such as goal-setting and action-planning, and patient motivation were noted as factors that may have reduced impacts.^30,38,41^

Studies showing a positive intervention effect suggest that improvements can be maintained with strategies, such as ongoing patient follow-up, patient feedback, individualised care plans, recording of lifestyle goals in the patient medical record, and booster training.^31,33,36,37,41,44,45^

## Discussion

### Summary

The key finding of this systematic review is that since 2013, the scarcity of studies that assess the impact on patient outcomes of training primary care clinicians in patient self-management of chronic conditions has been somewhat addressed. However, the generalisability of results is limited and it is not clear which intervention aspects work best.

The updated systematic review showed more evidence is now available with 18 articles in 5 years from September 2013 to August 2018 from the 4284 abstracts located. Twelve of the 18 articles showed a difference between groups, indicating that training health professionals in general practice to support their patients’ self-management activities results in improved patient outcomes. Seven of these were considered to have a low risk of bias, and overall nine were rated as moderate or strong on the quality assessment.

All educational interventions with health professionals in these articles spanned a range of techniques and modalities, and many incorporated multiple interventions including patient components. Several challenges, including that complex interventions may not be delivered as planned and are difficult to assess,^31,32,36,44^ often owing to workload implications,^31,36,40^ were found to be limiting factors. Patient-centred care appeared to increase the effectiveness of educational intervention with healthcare professionals in primary care.^32,35,42,46^ Some studies reported multiple follow-up time points in one article,^30,38,40,41,44^ while others reported these separately.^31,33,36,37,45^ Studies showing a positive intervention effect suggest that improvements can be maintained.^31,33,36,37,41,44,45^

### Strengths and limitations

The systematic review was limited to articles where educational interventions for patient self-management with health professionals in primary care were undertaken and the resultant patient outcomes were measured. Differences in terminology and concepts could have resulted in some articles not being located or included; however, the scope and criteria were clearly detailed.

Only articles in English were included, which could lead to reporting and language bias. The quality of studies varied, which could have introduced biases that can lead to over- or under-estimation of intervention effectiveness. Seven of the 18 included articles did not follow intention-to-treat analysis, which could induce attrition bias.

### Comparison with existing literature

Challenges to the delivery of such multifaceted programmes in primary care were identified by many of the studies. While some were related to research integrity, others were related to the feasibility of implementing interventions, particularly complex or prolonged interventions, in the real-world setting, as discussed elsewhere in the literature.^23^

Patient-centred care was identified as improving intervention effectiveness and is supported by findings that highlight the impact of good communication and trust,^47,48^ and the importance of personalised support and goal-setting,^49^ suggesting that empowerment-based strategies result in increased and longer self-efficacy improvement.^7^ This concept of patient-centred care supports the findings of the previous systematic review in relation to the role of motivational interviewing.^24^

### Implications for research and practice

There is a need to distill what methods work best in different settings and for different patients.^50,51^ Incorporating the phases of transformation that individuals are in should be incorporated into future studies to enhance this understanding.^8,23,52^

Patient empowerment represents a challenge for healthcare professionals,^7^ and hence further research needs to ensure the contextual element is captured,^8^ and practice needs to find ways to overcome the real-world limits.^7,23,48,51,53^ Whole health system changes^48,51,54^ and the use of information and communication technology (ICT) are recommended.^48,54^

It has been recommended elsewhere, and is supported here, that treatment integrity and fidelity data should be reported in all behaviour change studies.^23,53^

Patient self-management support is recognised to be an effective component of comprehensive integrated chronic disease management. However, more high-quality research is needed on what methods work best, for which patients, and for what clinical conditions in the primary care setting. The practical implications of training healthcare professionals require specific attention.

## Registration

PROSPERO registered protocol: CRD42013004418.
